# Continuous *de novo* biosynthesis of haem and its rapid turnover to bilirubin are necessary for cytoprotection against cell damage

**DOI:** 10.1038/srep10488

**Published:** 2015-05-20

**Authors:** Taka-aki Takeda, Anfeng Mu, Tran Tien Tai, Sakihito Kitajima, Shigeru Taketani

**Affiliations:** 1 Department of Biotechnology, Kyoto Institute of Technology, Kyoto 606-8585, Japan

## Abstract

It is well known that haem serves as the prosthetic group of various haemoproteins that function in oxygen transport, respiratory chain, and drug metabolism. However, much less is known about the functions of the catabolites of haem in mammalian cells. Haem is enzymatically degraded to iron, carbon monoxide (CO), and biliverdin, which is then converted to bilirubin. Owing to difficulties in measuring bilirubin, however, the generation and transport of this end product remain unclear despite its clinical importance. Here, we used UnaG, the recently identified bilirubin-binding fluorescent protein, to analyse bilirubin production in a variety of human cell lines. We detected a significant amount of bilirubin with many non-blood cell types, which was sensitive to inhibitors of haem metabolism. These results suggest that there is a basal level of haem synthesis and its conversion into bilirubin. Remarkably, substantial changes were observed in the bilirubin generation when cells were exposed to stress insults. Since the stress-induced cell damage was exacerbated by the pharmacological blockade of haem metabolism but was ameliorated by the addition of biliverdin and bilirubin, it is likely that the de novo synthesis of haem and subsequent conversion to bilirubin play indispensable cytoprotective roles against cell damage.

The biosynthesis of haem requires eight enzymes, whereas its catabolism requires three. The first and last three steps in haem biosynthesis take place in the mitochondria (Supplementary Fig. S1). At the first step, 5-aminolevulinic acid (ALA) synthase catalyses the condensation of glycine and succinyl-CoA to form ALA^1^,^2^. Ferrochelatase is the terminal enzyme in haem biosynthesis, catalysing the insertion of ferrous ions into protoporphyrin IX (PPIX) to form haem^3^,^4^. The synthesised haem is transported outside of mitochondria and utilised for the maturation of haem proteins. Haem metabolism is known to be regulated at several steps and is additionally dependent on the control of the circadian rhythm, hormones, and oxidative stress. Moreover, haem itself regulates its own homeostasis, cell differentiation, and cell proliferation^5^,^6^,^7^. However, little is known regarding the link between haem and other metabolic processes.

Bilirubin is the end product of haem degradation. It is produced by the action of haem oxygenase (HO), which degrades haem to produce biliverdin, iron, and carbon monoxide (CO)^8^,^9^. Lastly, cytosolic biliverdin reductase produces bilirubin, which is excreted after conjugating with glucuronate in the liver. HO (known as HO-1 and HO-2) serves as a regulator to maintain the intracellular level of haem. Iron produced by HO is reutilised as functional iron in proteins^10^,^11^,^12^. Bilirubin possesses antioxidant properties^13^,^14^. Water-insoluble unconjugated bilirubin bound to albumin is transferred to hepatocytes and taken up by the action of multiple transport systems^13^,^14^. After glucuronidation of bilirubin by hepatic enzymes, conjugated bilirubin is excreted to bile. Disrupted regulation of the hepatobiliary transport system has been shown to lead to jaundice in various hepatic disorders^14^,^15^. Although bilirubin in bile is reported to be derived predominantly from haemoglobin of senescent erythrocytes via the hepatic metabolic pathway^15^, the generation and transport of bilirubin in peripheral tissues have not been reported. In addition, CO can be related to cytoprotection against oxidative damage via reaction with stress-inducible HO-1^16^,^17^. Therefore, the physiological roles of the induction of HO-1 seem to be the preservation of tissue integrity against oxidative stress, contribution to the modulation of inflammatory responses *in vivo*, and acting as a tissue defence mechanism^16^,^17^,^18^. However, the precise mechanism by which HO-1 mediates its protective functions remains unknown.

Recently, it was found that the eel fluorescent protein UnaG binds unconjugated bilirubin with high affinity^19^. The quantification of bilirubin in tissues and serum has been suggested as a useful application for UnaG. We examined the generation of bilirubin in human cells using UnaG, finding that all of the cell types examined continuously generate and export bilirubin, beginning with haem biosynthesis. In addition, cells maintain the flow of haem metabolism and produce and export the end product bilirubin. This continuous stream of haem metabolism is required for protection from cellular stresses and the maintenance of cellular homeostasis.

## Results

### Biosynthesis of bilirubin in the cells and its export into medium

**Figure 1:**
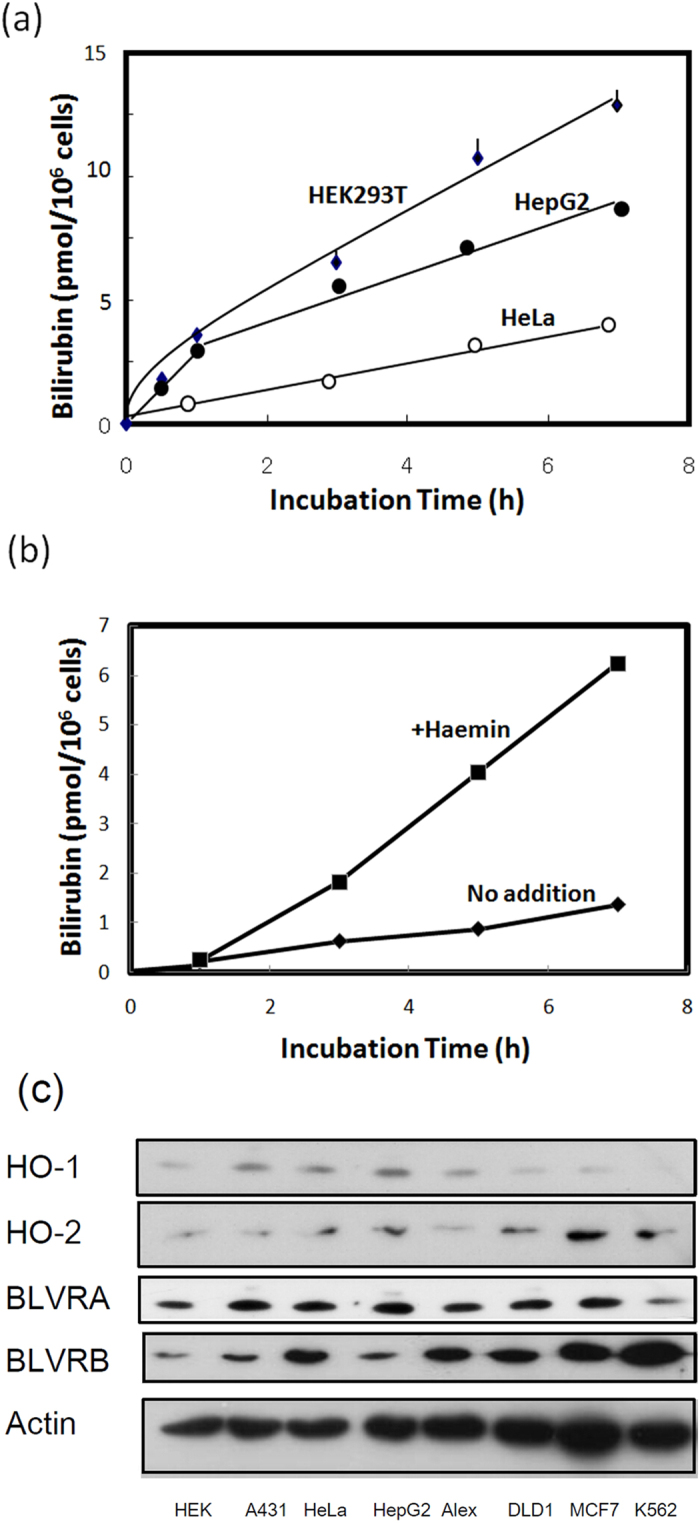
The production and export of bilirubin in human cells. (**a**) Time course of bilirubin released from cells in culture medium. HEK293T, HepG2, and HeLa cells were incubated with FCS-free VP-SFM medium plus 2.0 mg/ml bovine serum albumin (BSA). At the indicated time, aliquots of the media were withdrawn and incubated with recombinant his-tagged UnaG (2 μg/ml) for 1 h, followed by shaking with nickel ion beads for 30 min. After washing the beads twice with 10 mM Tris-HCl, pH 7.5, containing 150 mM NaCl (TBS), UnaG was eluted with TBS containing 300 mM imidazole. The fluorescence in elutes was examined by fluorospectrophotometry. (**b**) The production of bilirubin in K562 cells. K562 cells were incubated without or with 20 μM haemin, under conditions similar to those above. Bilirubin in the medium was estimated using fluorophotometry. (**c**) Immunoblot analysis of proteins involved in the generation of bilirubin. The cellular proteins of the indicated human cells were analysed by SDS-PAGE and electroblotted onto a PVDF membrane. The immunoblotting was performed with primary antibodies for HO-1, HO-2, BLVRA, BLVRB, and actin.

**Figure 2:**
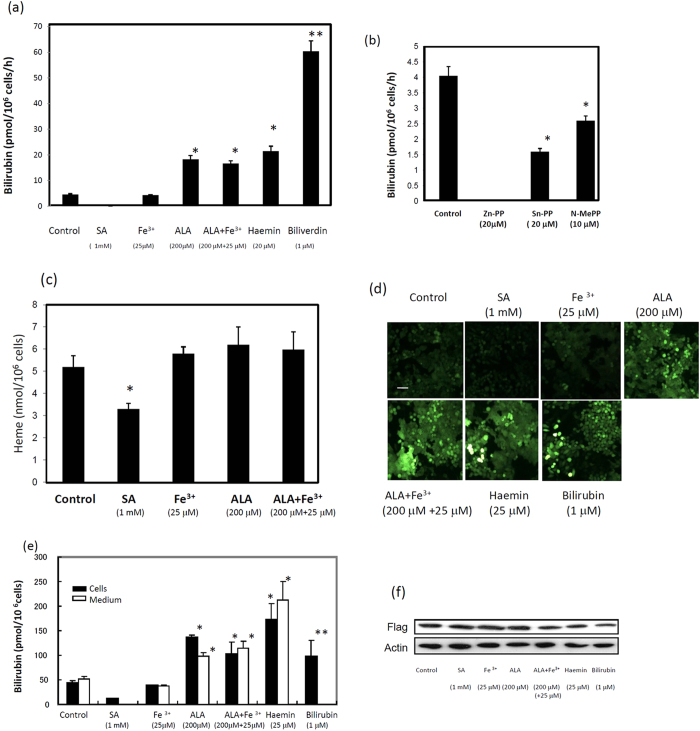
Changes in the production of bilirubin and levels of intracellular haem by treatment with a haem biosynthesis inhibitor, SA, or bilirubin precursors. (**a**) Bilirubin in culture medium. After incubation of HEK293T cells for 7 h, the culture medium was incubated with the recombinant UnaG and then with nickel beads. After washing the beads, the proteins were eluted. Bilirubin was estimated using fluorophotometry. The data are expressed as the mean ± standard deviation (SD) (n = 4 for each group). *, *P* < 0.005 and **, *P* < 0.001 vs. control. (**b**) Effect of the inhibitors. The cells were incubated in the presence of the indicated inhibitors for 16 h. The level of bilirubin in the medium was estimated as above. (**c**) Haem content. HEK293T cells were incubated in the FCS-free VP-SFM medium in the presence of the indicated compounds for 16 h. The intracellular level of haem was estimated by a haem-detection assay. The data are expressed as the mean ± SD (n = 4 for each group). *, *P* < 0.01 vs. control. (**d**) Microscopic observation. UnaG-expressing HepG2 transfectants were incubated in the FCS-free medium in the presence of the indicated compounds for 16 h. The living cells were directly observed by fluoromicroscopy. Bar: 20 μm. (**e**) Intra- and extracellular levels of bilirubin. HepG2 cells treated as above were collected, washed twice with PBS, and lysed with TBS containing 0.3% Tween 20. The fluorescence of the lysates and medium was examined by spectrophotometry. The data are expressed as the mean ± SD (n = 4 for each group). *, *P* < 0.01 and **, *P* < 0.05 vs. control. (**f**) Immunoblot analysis of UnaG. UnaG-expressing HepG2 transfectants were treated with the indicated compounds for 16 h. The cellular proteins were analysed by SDS-PAGE, followed by immunoblotting. Anti-flag and anti-actin were used as the primary antibodies.

**Figure 3:**
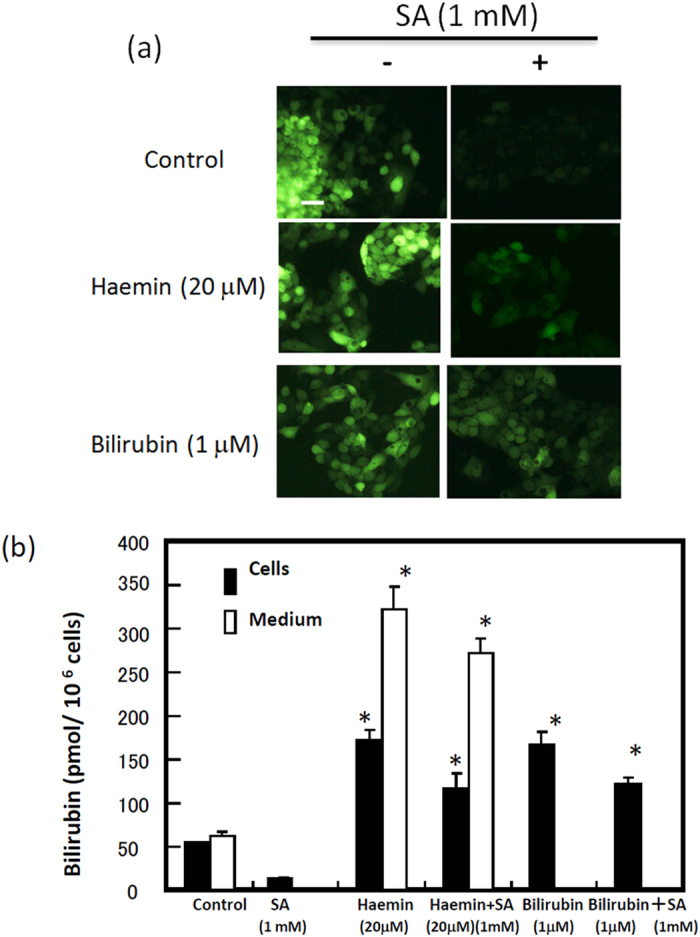
Effect of succinyl acetone (SA), haemin, or bilirubin on the generation of bilirubin in HepG2 cells expressing UnaG. (**a**) Microscopic observation. The cells were incubated in BSA-containing VP-SFM medium without or with 1 mM SA for 16 h. The cells were also treated with 20 μM haemin and 1 μM bilirubin. The fluorescence in living cells was examined by fluoromicroscopy. (**b**) Intra- and extracellular levels of bilirubin. The cells were incubated as above. Bilirubin levels in the cells and in the culture medium were examined. The data are expressed as the mean ± SD (n = 3 for each group). *, *P* < 0.005.

UnaG was found to bind to unconjugated bilirubin, with this complex becoming fluorescent^19^. To examine the generation of bilirubin in human cells, we first measured the level of bilirubin in cell culture using recombinant UnaG (Supplementary Fig. S2). Because foetal calf serum (FCS) contains bilirubin (7-10 pmol/ml), we used bilirubin-free synthetic medium VP-SFM instead of FCS-containing DMEM medium to examine the level of bilirubin. The level of bilirubin in the media of HEK 293T, HeLa and HepG2 cells increased linearly up to 5 h (Fig. 1a). The amounts of bilirubin produced by DLD-1, Alexander, A431, and MCF7 cells were similar to those produced by HeLa cells. Detectable but low amounts of bilirubin were released from human erythroleukaemia K562 cells in a time-dependent manner (Fig. 1b). Treatment of K562 cells with haemin resulted in an increase in the production of bilirubin. Immunoblotting revealed that certain enzymes, including HO-1, HO-2, biliverdin reductase A (BVRA), and biliverdin reductase B (BVRB), which were involved in the formation of bilirubin from haem, were ubiquitously expressed in these cells, with the exception that HO-1 was not expressed in K562 cells (Fig. 1c). These results indicated that all of the cells examined constantly generate bilirubin. When HEK293T cells were treated with ALA, haemin, or biliverdin, the level of bilirubin in the medium was markedly increased (Fig. 2a). Conversely, no bilirubin in the medium was detected by the treatment of cells with succinyl acetone (SA), a specific inhibitor of ALA dehydratase. When cells were treated with Sn-protoporphyrin (Sn-PP), an inhibitor of HO, or *N-*methyl protoporphyrin (N-MePP), an inhibitor of ferrochelatase, bilirubin levels in the medium were decreased (Fig. 2b). Zn-protoporphyrin (Zn-PP) completely abolished bilirubin formation. We also examined the intracellular level of haem in HEK cells. As shown in Fig. 2c, haem was slightly increased by ALA or iron citrate treatment while SA treatment decreased its level. Next, we examined the generation of bilirubin with HepG2 and HeLa cells that stably expressed UnaG (Supplementary Fig. S3). The cells in bilirubin-free medium exhibited fluorescence (Fig. 2d; Supplementary Fig. S4). The intensity of the fluorescence in cells and medium was increased by the treatment with ALA, haemin, and bilirubin (Fig. 2e), whereas SA decreased the fluorescence. The level of UnaG in HepG2 cells expressing UnaG was not changed by any of the treatments (Fig. 2f). We next compared bilirubin formation with or without SA. This treatment decreased the intracellular level of bilirubin in the medium, even following the addition of haemin or bilirubin (Fig. 3a,b), indicating that *de novo*-synthesised bilirubin was preferentially utilised as a ligand of UnaG.

### HO-1 induction by sodium arsenite, cadmium chloride, and diethyl malate (DEM) did not increase the formation of bilirubin

**Figure 4:**
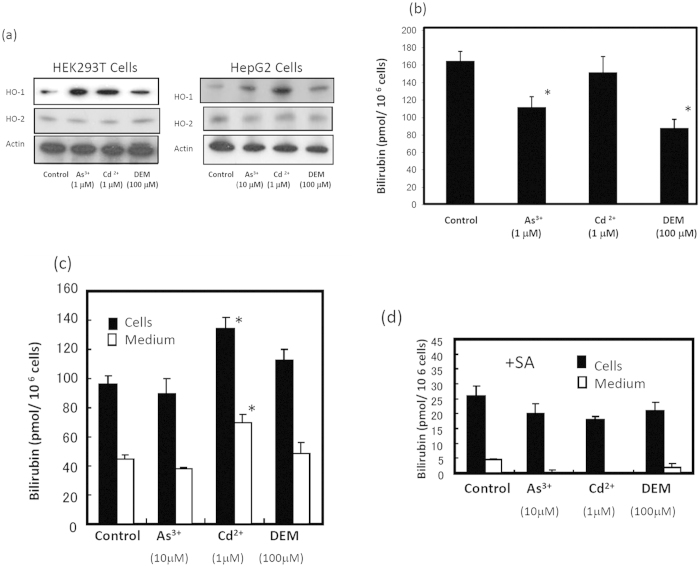
Generation of bilirubin in arsenite-, cadmium-, and diethyl malate (DEM)-treated HEK 293T and HepG2 cells. (**a**) Immunoblot analysis. HEK293T or HepG2 cells were treated in FCS-free medium with 1 μM sodium arsenite, 1 μM cadmium chloride, or 100 μM DEM for 16 h. The proteins in the cells were analysed by SDS-PAGE and electroblotted on a PVDF membrane. Immunoblotting was performed with anti-HO-1, anti-HO-2, and anti-actin as the primary antibodies. (**b**) Bilirubin in the culture medium. After treatment of HEK293T cells as above, bilirubin levels in the medium were examined using the recombinant UnaG. The data are expressed as the mean ± SD (n = 4 for each group). *, *P* < 0.01. (**c**) Bilirubin in UnaG-expressing HepG2 cells upon exposure to stress insults. HepG2 cells expressing UnaG in FCS-free VP-SFM medium were incubated with 10 μM sodium arsenite, 1 μM cadmium chloride, or 100 μM DEM for 16 h. The cells were collected, washed with PBS, and lysed. The fluorescence in cell lysates was measured by fluorospectrophotometry. The level of bilirubin in the culture medium was also estimated. *, *P* < 0.01. (**d**) Bilirubin in SA-treated HepG2 cells. The cells were also treated with chemicals as above in the presence of 1 mM SA for 16 h. Intra- and extracellular bilirubin levels were determined. The data are expressed as the mean ± SD (n = 4 for each group).

Although it is known that HO-1 induction by the HO-1 inducer haemin increases intracellular iron^1^,^20^, it is not clear whether the non-haem inducer of HO-1 affects bilirubin formation. When we treated HEK293T cells with sodium arsenite, cadmium chloride, and diethyl malate (DEM), the level of HO-1 increased (Fig. 4a). The treatment of HEK cells with these insults either decreased or slightly increased the formation of bilirubin, indicating that no significant increase in the formation occurs under stress-induced conditions (Fig. 4b). HepG2 cells expressing UnaG in all tested treatments also showed no significant change in the bilirubin formation intra- or extracellularly, except for a slight increase in bilirubin in cadmium ion-treated cells (Fig. 4c). Furthermore, SA treatment of HepG2 cells diminished bilirubin in the medium even in the presence of the inducers (Fig. 4d). These results indicate that the turnover or degradation of haem within haem proteins cannot be accelerated under stress conditions.

### Export of bilirubin from cells via transporters

**Figure 5:**
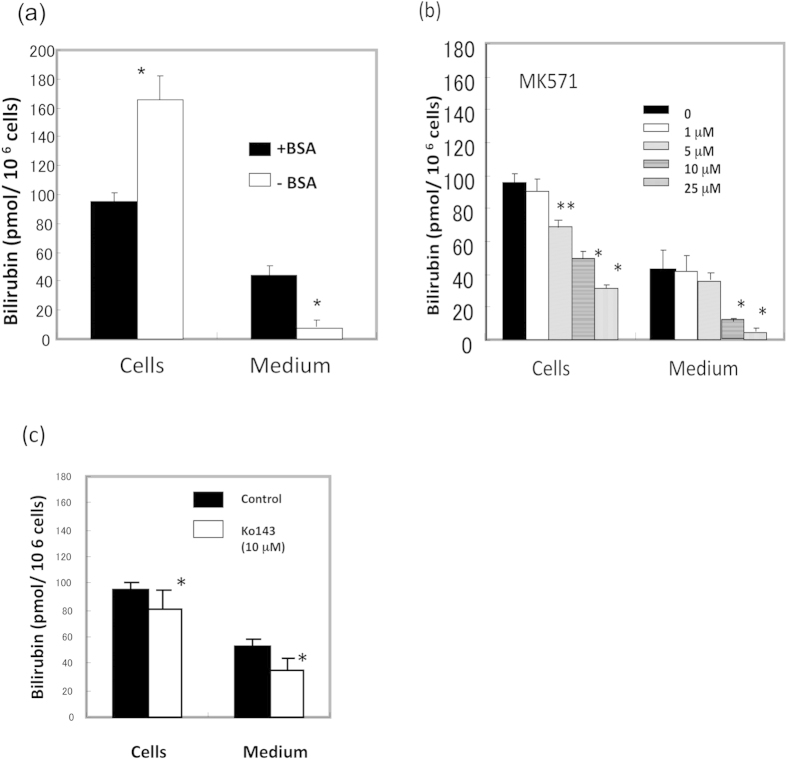
Effect of inhibitors of ABC-type transporters on the export of bilirubin. (**a**) Effect of BSA on the export of bilirubin. HepG2 cells expressing UnaG were incubated in FCS-free VP-SFM medium without or with 2.0 mg/ml BSA for 16 h. The levels of bilirubin in the cells and culture media were estimated as above. The data are expressed as the mean ± SD (n = 3 for each group). *, *P* < 0.01 vs. control (+BSA). (**b**) Effect of MK571. HepG2 cells in BSA containing FCS-free VP-SFM medium were treated with the indicated concentrations of MK571 for 16 h. The levels of bilirubin in the cells and culture media were estimated as above. (**c**) Effect of Ko143. Bilirubin levels in the cells and the media were examined.

To examine how cells export bilirubin into the medium, cells were incubated in the absence or presence of bovine serum albumin (BSA). The presence of BSA led to a marked increase in the release of bilirubin (Fig. 5a), indicating that albumin is necessary to export bilirubin from cells. We next treated cells with MK571, an inhibitor of ABC-type pump protein MRP2/3. The level of bilirubin in the medium decreased depending on the concentration of MK571 (Fig. 5b). Interestingly, intracellular bilirubin was concomitantly decreased. Another inhibitor of the pump protein ABCG2, Ko143, slightly decreased the level of bilirubin in the medium and cells (Fig. 5c). These results indicate that pump proteins, including MRP2/3, can be involved in the export of bilirubin and that inhibition of the export of bilirubin by these inhibitors suppressed *de novo* synthesis of bilirubin. Separately, when we examined the level of protoporphyrin and haem in MK571- or Ko143-treated cells, an accumulation of protoporphyrin and a decrease of haem were observed (data not shown). These results suggest that these inhibitors may block the transport of porphyrin or haem in mitochondria.

### The cytoprotective roles of biliverdin and bilirubin in the HO reaction

**Figure 6:**
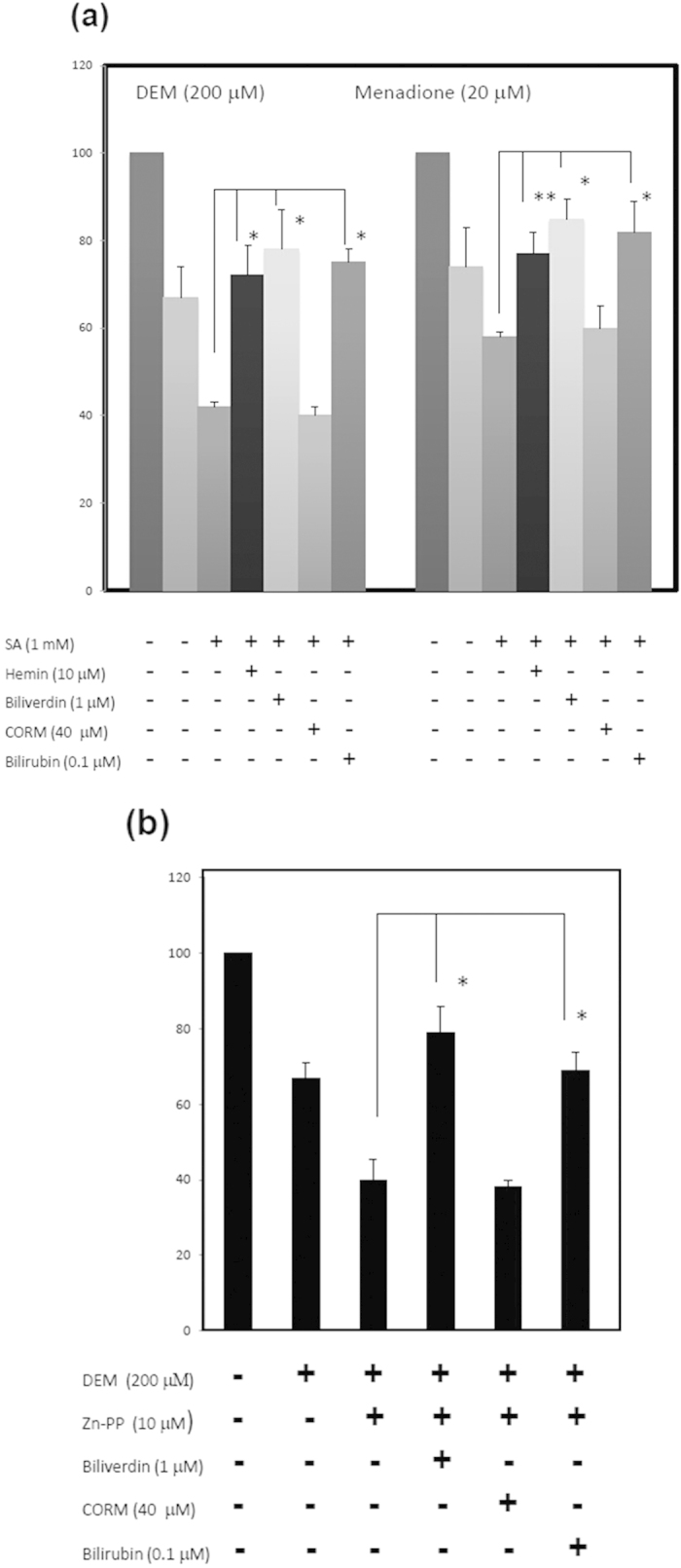
The increase of oxidative damage by inhibition of haem metabolism and the restoration by biliverdin and bilirubin. (**a**) The increase of cell damage with SA and restoration by biliverdin and bilirubin. HEK293T cells were incubated with 200 μM DEM and 20 μM menadione with or without 1 mM SA. At the beginning of incubation, 10 μM haemin, 40 μM CORM2, 1 μM biliverdin, or 0.1 μM bilirubin was also added to the culture medium. After 24 h of incubation, an MTT assay was carried out to evaluate cell damage. The data are expressed as the mean ± SD (n = 6 for each group). *, *P* < 0.01 and **, *P* < 0.05 vs. treatment with DEM plus SA. (**b**) Effect of Zn-protoporphyrin (Zn-PP), biliverdin, and bilirubin on oxidative damage. HEK293T cells were treated with chemicals as above, except for the use of 10 μM Zn-PP instead of the addition of SA. Cell damage was examined by MTT assay. Data are expressed as the mean ± SD (n = 5 for each group). *, *P* < 0.01.

To examine the physiological roles of the continuous intracellular generation of haem and its rapid turnover to bilirubin, cells were exposed to damage insults such as menadione and DEM^21^,^22^. A 3-(4,5-dimethylthiazol-2-yl)-2,5-diphenyltetrazolium bromide (MTT) assay was performed to evaluate cell damage. When HEK293T cells were treated with DEM and SA in combination, cell death was increased compared with that upon treatment with DEM alone (Fig. 6a). The cessation of haem biosynthesis leads to sensitivity to oxidative damage. The addition of 10 μM haemin to DEM- and SA-treated cells led to a restoration of cell viability. Higher concentrations of haemin (20-50 μM) induced cytotoxicity (data not shown). We then incubated DEM- and SA-treated cells with the reaction product of HO. Cell death was decreased compared with that upon the treatment without biliverdin, while the CO-forming chemical tricarbonyldichlororuthenium (II) dimer (CORM2) did not mitigate the cell damage. Bilirubin added exogenously at low concentrations (0.1 μM) resulted in the prevention of DEM- and SA-induced cell death. The cell damage caused by menadione plus SA was also reduced by biliverdin and bilirubin but not by CORM. The treatment of cells with DEM in the presence of Zn-PP increased cell death, and the addition of haemin slightly increased the number of living cells (Fig. 6b), suggesting that the reaction of HO contributes to the resistance to cell damage. The cell damage caused by DEM with Zn-PP was not restored by CORM2, but restoration was observed following treatment with biliverdin. These results suggested that the flow of the production of biliverdin and bilirubin by the HO reaction in cells has a cytoprotective role.

## Discussion

In this study, we first demonstrated that various cells constantly produce and degrade haem. Bilirubin is ultimately generated and is subsequently exported from the cells into the medium. To eliminate the influx of bilirubin into the medium, we used bilirubin-free medium, finding that all of the cells examined constantly produced bilirubin. All cells examined express enzymes involved in haem catabolism (Fig. 1c). Erythroleukaemia K562 cells produced approximately one-tenth of the bilirubin produced by HEK 293 and HepG2 cells. K562 cells do not express HO-1, but HO-2 degrades haem to form biliverdin, which is reduced to bilirubin by biliverdin reductase (BVR). Thus, even erythroid cells, which utilise haem in large amounts for haemoglobin synthesis, produce bilirubin through the degradation of haem. Kumagai *et al.* (2013)^19^ reported that bilirubin is amply present in whole brain of mouse embryo (E16.5) and HeLa cells as determined by UnaG fluorescence. An early study^23^ used a pulse-labelled experiment with radioactive ALA or iron, reporting that there was rapid degradation of newly synthesised haem in rat liver and isolated hepatocytes. From these findings, we concluded that mammalian cells constantly synthesise bilirubin from the initial step of haem biosynthesis through a haem-metabolising pathway.

Exposure of cells to non-haem stress inducers, including arsenite, cadmium ions, and DEM, resulted in the induction of HO-1 expression, but only small changes were shown in the production of bilirubin (Fig. 4b,c). Furthermore, treatment with SA led to complete cessation of bilirubin production under the arsenite-, cadmium-, and DEM-induced stress conditions. This observation indicates that the induction of HO-1 was not always coupled to the degradation of the haem moiety of haem protein to protect the cells from oxidative stress. Similar observations were made by Shetefel *et al.*^24^; namely, that the non-haem inducer arsenite induced the expression of HO-1 in mouse macrophage RAW264.7 cells but did not alter the cellular metabolism of iron. These authors also found that cells stably overexpressing HO-1 were protected from oxidative stress, but ferritin synthesis did not increase. An *in vivo* study^25^ showed that the urinary level of bilirubin in arsenite-administered mice was modest to strong following the induction of hepatic HO-1. Urinary bilirubin quickly returned to the basal level, although hepatic HO-1 continued to be expressed at a high level. Therefore, the induction of HO-1 is not related to the degradation of haem under oxidative stress, but other processes may occur in response to cellular stresses. In contrast, it is thought that increased HO-1 results in acceleration of the degradation of intracellular haem and the subsequent change in the intracellular status of iron^26^. These events may be related to cytoprotection against oxidant stress. In fact, it was observed that the overexpression of HO-1 altered the intracellular distribution of iron, leading to a cytoprotective effect against hydrogen peroxide^18^. On the basis of these observations, there is not always a correlation between the induction of HO-1 and the degradation of haem and iron metabolism. However, the regulation of haem and iron metabolism is cell-specific. Considering that the expression of catalytically inactive HO-1 or HO-2 still resulted in a cytoprotective effect against oxidative stress^27^,^28^, native HO can be said to be sufficiently cytoprotective; alternatively, the cytoprotective function of HO can be separated from the extent of haem degradation by the HO reaction.

The principal metabolic route for the removal of excess bilirubin is conjugation with glucuronic acid in the liver and elimination in bile, a reaction catalysed by hepatic UDP-glucuronyl transferase. Previous studies^13^,^14^,^15^ reported that several types of ABC-type pump proteins, including MRP2/3 and ABCG2 in hepatocytes, are involved in the export of conjugated or unconjugated bilirubin to bile. Most studies on the production and transport of bilirubin were performed in hepatocytes, but not in non-hepatocytes. The present study demonstrated that the ABC-type transport protein family, including MRP2/3 and ABCG2 at the plasma membrane, can be involved in the extracellular export of bilirubin in HepG2 cells given that the inhibitors of the pump proteins MK571 and Ko143 decreased bilirubin in medium. The fact that the substrate specificities of MK671 and Ko143 are not specific for MRP2/3 and ABCG2^29^, respectively, indicates that multiple exporters can be involved in the export of bilirubin. It is notable that the level of bilirubin in MK571- or Ko143-treated cells decreased in a manner dependent on the decrease of bilirubin in the medium. These results suggest that feedback regulation exists at some step in the haem metabolic pathway, functioning to stop the export of bilirubin. Furthermore, an accumulation of protoporphyrin was observed in MK571-treated cells, indicating that this drug inhibited the final step of haem biosynthesis or mitochondrial haem transporter Flvcr1b^30^.

Many studies^8^,^10^,^31^ have shown the cytoprotective effect of the HO reaction or its products CO, biliverdin, and bilirubin. We have found that haem is constantly synthesised, degraded, and finally converted to bilirubin. In addition, cells constantly generate CO, biliverdin, and bilirubin at the basal level (Figs. 2,3). Therefore, to examine the physiological significance of the continuous turnover of haem (i.e., the haem stream), cells were exposed to the oxidative reagents DEM and menadione. Cell damage was increased by treatment with SA, indicating that haem synthesis is necessary for protection against oxidative damage. The addition of 10 μM haemin partially reversed the cell damage, indicating that the haem stream is essential for protecting against oxidative damage. Conversely, exogenously added haemin at excess amounts caused cell damage. Thus, haem is a double-faced molecule: at physiological levels, it performs its essential functions, whereas excess levels of haem result in oxidative stress or cell injury. In the presence of Zn-PP, a competitive inhibitor of the HO reaction, the cells became sensitive to damage, suggesting that HO reaction products are possible protectors against the damage.

Lastly, biliverdin and bilirubin, but not CO, mitigated the cell damage. Bilirubin at a low concentration (100 nM) was effective at preventing cell damage, but did not have an effect at higher concentrations (1-10 μM). In the culture medium containing 10% FCS, the concentration of bilirubin corresponded to approximately 1 nM bilirubin, which is insufficient to prevent oxidative stress. However, considering that another investigation^32^ showed that the treatment of cells with 1-20 μM bilirubin induced HO-1 via activation of the stress-responsive transcription factor Nrf2, the use of bilirubin at higher concentrations caused cell stress. On the basis of our findings that bilirubin in SA-treated cells was not completely complemented by exogenously added bilirubin (Fig. 3a,b), it seems that *de novo*-generated bilirubin in cells is much more effective at preventing cell damage than exogenously added bilirubin. Therefore, it is possible that the conversion of biliverdin to bilirubin by BVR may play an important role in the cytoprotection against oxidative damage. In this regard, BVR regulated by the substrate biliverdin becomes a transcription factor that is a critical regulator of innate immune responses resulting from acute insult and injury^33^,^34^.

Although we did not observe a protective effect of CO against DEM- and menadione-induced cell damage, it is known that CO is beneficial for cell survival *in vitro* and *in vivo*^16^,^31^. The present data indirectly demonstrate that CO is constantly formed by the HO reaction and suggest that CO binds to unknown molecules, such as reduced haem proteins. This can be inferred from the fact that CO shows high affinity for reduced forms of haemoproteins, thereby stabilising them or preventing their oxidative denaturation.

It is well known that approximately 80% of bilirubin formed each day is derived from the degradation of erythrocyte haemoglobin^35^. The remaining 20% is from insufficient erythropoiesis in bone marrow and the degradation of other haem proteins. Studies on bilirubin metabolism have been mainly conducted with human and animal livers^14^,^15^. The present data provide evidence that bilirubin in peripheral cells is directly generated via the haem stream pathway and not always via haem proteins. Earlier studies^36^,^37^ have shown that when radiolabelled ALA was injected into rats, radioactive bilirubin appeared in bile within 15 min. This bilirubin may have been derived from a rapidly turning over pool of cytosol in hepatocytes that is degraded without incorporation into haem proteins. The present results demonstrate that newly synthesised haem is continuously turned over to bilirubin in a variety of tissues. Bilirubin is exported extracellularly, bound to albumin, and transported to the liver through the circulatory system. The liver has the capacity to take up a very large amount of bilirubin every second. In this regard, patients with haemolytic anaemia suffer hyperbilirubinaemia due to an elevation of unconjugated bilirubin in serum, which can be caused by the overproduction of bilirubin^13^,^15^. A decrease of bilirubin clearance by hepatocytes may cause the accumulation of unconjugated bilirubin, but this is very rare^38^,^39^. Possible defects in bilirubin transporters remain to be identified and may be linked to unidentified severe diseases. The normal liver expresses a large number of multiple transporters, including anion organic acid transporters, which can eliminate a great deal of bilirubin from the circulation and by conjugation with glucuronate.

## Methods

### Materials

Restriction endonucleases and DNA-modifying enzymes were purchased from Takara Co. (Tokyo, Japan) and Toyobo Co. (Tokyo, Japan). Antibodies for flag-tag, BVRA, and BVRB were products of Sigma (St. Louis, MI). The polyclonal antibody for actin (sc-1615) was obtained from Santa Cruz Biotechnology (Santa Cruz, CA). Antibodies for HO-1 and HO-2 were as previously described^12^. ALA, MK571, Ko143, and SA were purchased from Sigma Co. (St. Louis, MI). Sn-PP and Zn-PP were products of Porphyrin Products (Logan, UT). All other chemicals were of analytical grade.

### Construction of the UnaG expression plasmid

To construct a bacterial expression vector carrying UnaG, pcDNA3-flag-UnaG^19^ was digested with BamHI and EcoRI, and the isolated insert was ligated into the BamHI and *Eco*RI sites of pET32a (+) (Merck, Tokyo, Japan). Thus obtained pET-UnaG was transformed into the BL21 strain. UnaG was induced with 0.3 mM isopropyl thiogalactopyranoside (IPTG) and purified with nickel ion beads (Qiagen, Tokyo, Japan).

### Cell culture

Human epithelial cervical cancer HeLa cells, human hepatoma HepG2 cells, human epidermal carcinoma A431 cells, human breast cancer MCF7 cells, human hepatoma Alexander (PLC/PRF/5) cells, and human embryonic kidney HEK293T cells were grown in Dulbecco’s modified Eagle’s medium (DMEM) supplemented with 7% FCS, penicillin (100 units/ml), and streptomycin (100 μg/ml). Human colon cancer DLD-1 cells and human erythroleukemia K562 cells were grown in RPMI1640 medium containing 7% FCS and antibiotics. HEK293T cells were transfected with pcDNA3-flag UnaG^19^ using Lipofectamine 2000 (Invitrogen) and incubated for 16 h. For the establishment of stable transfection of HepG2 and HeLa cells, pcDNA3-flag UnaG (10 μg) was transfected with calcium phosphate into HepG2 or HeLa cells, as described previously^40^. For selection, G418 (Wako Chemicals, Tokyo, Japan) at a final concentration of 300 μg/ml was added to the culture medium. After 5 days, colonies of the G418-resistant cells were trypsinised, seeded in a 24-well tissue culture plate, and cultured in medium containing G418 (300 μg/ml). Individual clones were isolated and examined for the expression of UnaG by fluorescence microscopy. Four UnaG-expressing clones were obtained, mixed to avoid clonal variation, and maintained in DMEM containing 7% FCS and antibiotics. As control cells (Mock), HepG2 cells were transfected with pcDNA3.1 vector, and G418-resistant cells were isolated. Haem content in the cells was estimated colourimetrically using the haem-assay kit (Bioassay System, Hayward, CA).

### Fluorescence assay of bilirubin

The cells were cultured with bilirubin-free synthetic VP-SFM medium (Life Technologies, Grand Island, NY) containing 2.0 mg/ml BSA. The expression of UnaG in living cells was observed using a Nikon fluorescence microscope Model ECLIPSE E600 (Tokyo, Japan). Following cell lysis by sonication, the level of bilirubin bound to UnaG was examined by the fluorescence and measured using a spectrofluorometer, with excitation at 480 nm and emission by scanning from 500 to 550 nm^19^ . To estimate the level of bilirubin in the medium, the medium (1.0 ml) was incubated with the recombinant his-tagged UnaG (2 μg) for 1 h at 25 °C, after which the UnaG was trapped with Ni^2+^ beads (Qiagen, Tokyo, Japan). After washing with TBS, the UnaG was eluted with TBS containing 300 μM imidazole. The fluorescence in the eluates was examined.

### Immunoblotting

The lysates from the HEK293T cells were subjected to SDS-PAGE and electroblotted onto a poly(vinylidene difluoride) (PVDF) membrane (Bio-Rad Laboratories, Hercules, CA). Immunoblotting was carried out as described previously^12^,^40^.

### MTT assay

The cells were treated with chemical insults for 24 h and then pulsed with MTT (500 μg/ml) for 1 h; the resultant MTT formazan was solubilised with isopropanol. Absorbance at 590 nm was measured with a Microplate Reader NJ2001 (Japan InterMed. Co., Tokyo, Japan). Each experiment was carried out in quadruplicate or sextuplicate.

### Statistics

Results are shown as the mean ± SD and were analysed using unpaired Student’s t-test. All statistical analyses were considered significant at the level of *p* < 0.05 using GraphPad Prism software version 5.02 (GraphPad Software, Inc., CA)^12^,^40^.

## Author Contributions

T.T. performed most of the studies with UnaG (Figs. 1–5) and initiated the writing of the paper. A.M. performed the experiments for Fig. 5 and contributed to Figs. 2 and 3. T.T.T. performed the analysis of the MTT assay (Fig. 6). S.K. was involved in the maintenance and care of the HepG2 colonies. Lastly, S.T. designed, coordinated, supervised, and directed all of the studies described herein, performed the experiments shown Figs. S1-3, and wrote the manuscript.

## Additional Information

**How to cite this article**: Takeda, T.-a. *et al.* Continuous *de novo* biosynthesis of haem and its rapid turnover to bilirubin are necessary for cytoprotection against cell damage. *Sci. Rep.* **5**, 10488; doi: 10.1038/srep10488 (2015).

## Supplementary Material

Supporting Information

## References

[b1] Taketani S. Aquisition, mobilization and utilization of cellular iron and heme: endless findings and growing evidence of tight regulation. Tohoku J. Exp. Med. 205, 297–318 (2005). 15750326 10.1620/tjem.205.297

[b2] Sassa S. Regulation of the genes for heme pathway enzymes in erythroid and in non-erythroid cells. Int. J. Cell Cloning 8, 10–26 (1990). 2403580 10.1002/stem.5530080104

[b3] Sakaino M. *et al.* Dual mitochondrial localization and different roles of the reversible reaction of mammalian ferrochelatase. FEBS J. 276, 5559–5570 (2009). 19691493 10.1111/j.1742-4658.2009.07248.x

[b4] Crooks D. R., Ghosh M. C., Haller R. G., Tong W. H. & Rouault T. A. Posttranslational stability of the heme biosynthetic enzyme ferrochelatase is dependent on iron availability and intact iron-sulfur cluster assembly machinery. Blood 115, 860–869 (2010). 19965627 10.1182/blood-2009-09-243105 PMC2815515

[b5] Feng D. & Lazar M. A. Clocks, metabolism, and the epigenome. Mol. Cell 47, 158–167 (2012). 22841001 10.1016/j.molcel.2012.06.026 PMC3408602

[b6] Itoh R. *et al.* Imaging of heme/hemeproteins in nucleus of the living cells expressing heme-binding nuclear receptors. FEBS Lett. 587, 2131–2136 (2013). 23735699 10.1016/j.febslet.2013.05.036

[b7] Igarashi K. & Watanabe-Matsui M. Wearing red for signaling: the heme-bach axis in heme metabolism, oxidative stress response and iron immunology. Tohoku J. Exp. Med. 232, 229–253 (2014). 24681888 10.1620/tjem.232.229

[b8] Baranano D. E. & Snyder S. H. Neural roles for heme oxygenase: contrasts to nitric oxide synthase. Proc. Natl. Acad. Sci. U. S. A. 98, 10996–11002 (2001). 11572959 10.1073/pnas.191351298 PMC58673

[b9] Alam J., Igarashi K., Immenschuh S., Shibahara S. & Tyrrell R. M. Regulation of heme oxygenase-1 gene transcription: recent advances and highlights from the International Conference (Uppsala, 2003) on Heme Oxygenase. *Antioxid*. *Redox. Signal* . 6, 924–933 (2004). 15345152 10.1089/ars.2004.6.924

[b10] Origassa C. S. & Camara N. O. Cytoprotective role of heme oxygenase-1 and heme degradation derived end products in liver injury. World J. Hepatol. 5, 541–549 (2013). 24179613 10.4254/wjh.v5.i10.541 PMC3812456

[b11] Gozzelino R., Jeney V. & Soares M. P. Mechanisms of cell protection by heme oxygenase-1. Annu. Rev. Pharmacol. Toxicol. 50, 323–354 (2010). 20055707 10.1146/annurev.pharmtox.010909.105600

[b12] Ohgari Y. *et al.* Roles of porphyrin and iron metabolisms in the delta-aminolevulinic acid (ALA)-induced accumulation of protoporphyrin and photodamage of tumor cells. Photochem. Photobiol. 87, 1138–1145 (2011). 21668870 10.1111/j.1751-1097.2011.00950.x

[b13] McDonagh A. F., Palma L. A. & Lightner D. A. Blue light and bilirubin excretion. Science 208, 145–151 (1980). 7361112 10.1126/science.7361112

[b14] Sticova E. & Jirsa M. New insights in bilirubin metabolism and their clinical implications. World J. Gastroenterol. 19, 6398–6407 (2013). 24151358 10.3748/wjg.v19.i38.6398 PMC3801310

[b15] Erlinger S., Arias I. M. & Dhumeaux D. Inherited disorders of bilirubin transport and conjugation: new insights into molecular mechanisms and consequences. Gastroenterology 146, 1625–1638 (2014). 24704527 10.1053/j.gastro.2014.03.047

[b16] Simon T., Anegon I. & Blancou P. Heme oxygenase and carbon monoxide as an immunotherapeutic approach in transplantation and cancer. Immunotherapy 3, 15–18 (2011). 21524161 10.2217/imt.11.43

[b17] Hull T. D., Agarwal A. & George J. F. The mononuclear phagocyte system in homeostasis and disease: a role for heme oxygenase-1. Antioxid. Redox. Signal. 20, 1770–1788 (2014). 24147608 10.1089/ars.2013.5673 PMC3961794

[b18] Lanceta L., Li C., Choi A. M. & Eaton J. W. Haem oxygenase-1 overexpression alters intracellular iron distribution. Biochem. J. 449, 189–194 (2013). 22989377 10.1042/BJ20120936

[b19] Kumagai A. *et al.* A bilirubin-inducible fluorescent protein from eel muscle. Cell 153, 1602–1611 (2013). 23768684 10.1016/j.cell.2013.05.038

[b20] Taketani S., Kohno H., Yoshinaga T. & Tokunaga R. The human 32-kDa stress protein induced by exposure to arsenite and cadmium ions is heme oxygenase. FEBS Lett. 245, 173–176 (1989). 2924920 10.1016/0014-5793(89)80215-7

[b21] Wang X. *et al.* Cytoprotection of human endothelial cells from menadione cytotoxicity by caffeic acid phenethyl ester: the role of heme oxygenase-1. Eur. J. Pharmacol. 591, 28–35 (2008). 18573251 10.1016/j.ejphar.2008.06.017

[b22] Taketani S. *et al.* Induction in mouse peritoneal macrophages of 34 kDa stress protein and heme oxygenase by sulfhydryl-reactive agents. J. Biochem. 108, 28–32 (1990). 2229007 10.1093/oxfordjournals.jbchem.a123156

[b23] Lodola A. & Jones O. T. Evidence for a rapidly turned over pool of haem in isolated hepatocytes. FEBS Lett. 90, 250–254 (1978). 668888 10.1016/0014-5793(78)80379-2

[b24] Sheftel A. D., Kim S. F. & Ponka P. Non-heme induction of heme oxygenase-1 does not alter cellular iron metabolism. J. Biol. Chem. 282, 10480–10486 (2007). 17242398 10.1074/jbc.M700240200

[b25] Arthur D. M., Ng J. C., Lang M. A. & Abu-Bakar A. Urinary excretion of bilirubin oxidative metabolites in arsenite-treated mice. J. Toxicol. Sci. 37, 655–661 (2012). 22688006 10.2131/jts.37.655

[b26] Kovtunovych G., Eckhaus M. A., Ghosh M. C., Ollivierre-Wilson H. & Rouault T. A. Dysfunction of the heme recycling system in heme oxygenase 1-deficient mice: effects on macrophage viability and tissue iron distribution. Blood 116, 6054–6062 (2010). 20844238 10.1182/blood-2010-03-272138 PMC3031391

[b27] Kim Y. S. & Dore S. Catalytically inactive heme oxygenase-2 mutant is cytoprotective. Free Radic. Biol. Med. 39, 558–564 (2005). 16043027 10.1016/j.freeradbiomed.2005.04.009

[b28] Hori R. *et al.* Gene transfection of H25A mutant heme oxygenase-1 protects cells against hydroperoxide-induced cytotoxicity. J. Biol. Chem. 277, 10712–10718 (2002). 11786534 10.1074/jbc.M107749200

[b29] Paul S., Breuninger L. M., Tew K. D., Shen H. & Kruh G. D. ATP-dependent uptake of natural product cytotoxic drugs by membrane vesicles establishes MRP as a broad specificity transporter. Proc. Natl. Acad. Sci. USA. 94, 14976 (1997). 9556431 PMC56166

[b30] Chiabrando D. *et al.* The mitochondrial heme exporter FLVCR1b mediates erythroid differentiation. J. Clin. Invest. 122, 4569–4579 (2012). 23187127 10.1172/JCI62422 PMC3533534

[b31] Dolinay T., Choi A. M. & Ryter S. W. Heme Oxygenase-1/CO as protective mediators in cigarette smoke- induced lung cell injury and chronic obstructive pulmonary disease. Curr. Pharm. Biotechnol. 13, 769–776 (2012). 22201606 10.2174/138920112800399338

[b32] Qaisiya M., Coda Zabetta C. D., Bellarosa C. & Tiribelli C. Bilirubin mediated oxidative stress involves antioxidant response activation via Nrf2 pathway. Cell. Signal. 26, 512–520 (2014). 24308969 10.1016/j.cellsig.2013.11.029

[b33] Maines M. D. Biliverdin reductase: PKC interaction at the cross-talk of MAPK and PI3K signaling pathways. Antioxid. Redox. Signal. 9, 2187–2195 (2007). 17919068 10.1089/ars.2007.1805

[b34] Wegiel B. *et al.* Biliverdin inhibits Toll-like receptor-4 (TLR4) expression through nitric oxide-dependent nuclear translocation of biliverdin reductase. Proc. Natl. Acad. Sci. USA 108, 18849–18854 (2011). 22042868 10.1073/pnas.1108571108 PMC3219137

[b35] London I. M., West R., Shemin D. & Rittenberg D. On the origin of bile pigment in normal man. J. Biol. Chem. 184, 351–358 (1950). 15422003

[b36] Schwartz S. *et al.* Erythropoietic defects in protoporphyria: a study of factors involved in labelling of porphyrins and bile pigments from ALA- 3 H and glycine- 14 C. J. Lab. Clin. Med. 78, 411–434 (1971). 5092862

[b37] Grandchamp B., Bissell D. M., Licko V. & Schmid R. Formation and disposition of newly synthesized heme in adult rat hepatocytes in primary culture. J. Biol. Chem. 256, 11677–11683 (1981). 7298624

[b38] Martin J. F. *et al.* Abnormal hepatic transport of indocyanine green in Gilbert’s syndrome. Gastroenterology 70, 385–391 (1976). 814028

[b39] Gentile S., Persico M. & Tiribelli C. Abnormal hepatic uptake of low doses of sulfobromophthalein in Gilbert’s syndrome: the role of reduced affinity of the plasma membrane carrier of organic anions. Hepatology 12, 213–217 (1990). 2391064 10.1002/hep.1840120206

[b40] Sawamoto M. *et al.* The p53-dependent expression of frataxin controls 5-aminolevulinic acid-induced accumulation of protoporphyrin IX and photo-damage in cancerous cells. Photochem. Photobiol. 89, 163–172 (2013). 22862424 10.1111/j.1751-1097.2012.01215.x

