# Supplementary material for: Continuous de novo biosynthesis of haem and its rapid turnover to bilirubin are necessary for cytoprotection against cell damage
Source: Sci Rep. 2015 May 20;5:10488. doi: 10.1038/srep10488 (PMC4438432; doi:10.1038/srep10488)
Supplement: Supporting Information [file srep10488-s1.pdf]

Supplementary Information

Continuous *de novo* biosynthesis of haem and its rapid turnover to bilirubin are necessary for cytoprotection against cell damage

Taka-aki Takeda, Anfeng Mu, Tran Tien Tai, Sakihito Kitajima, and Shigeru Taketani
Department of Biotechnology, Kyoto Institute of Technology, Kyoto 606-8585, Japan

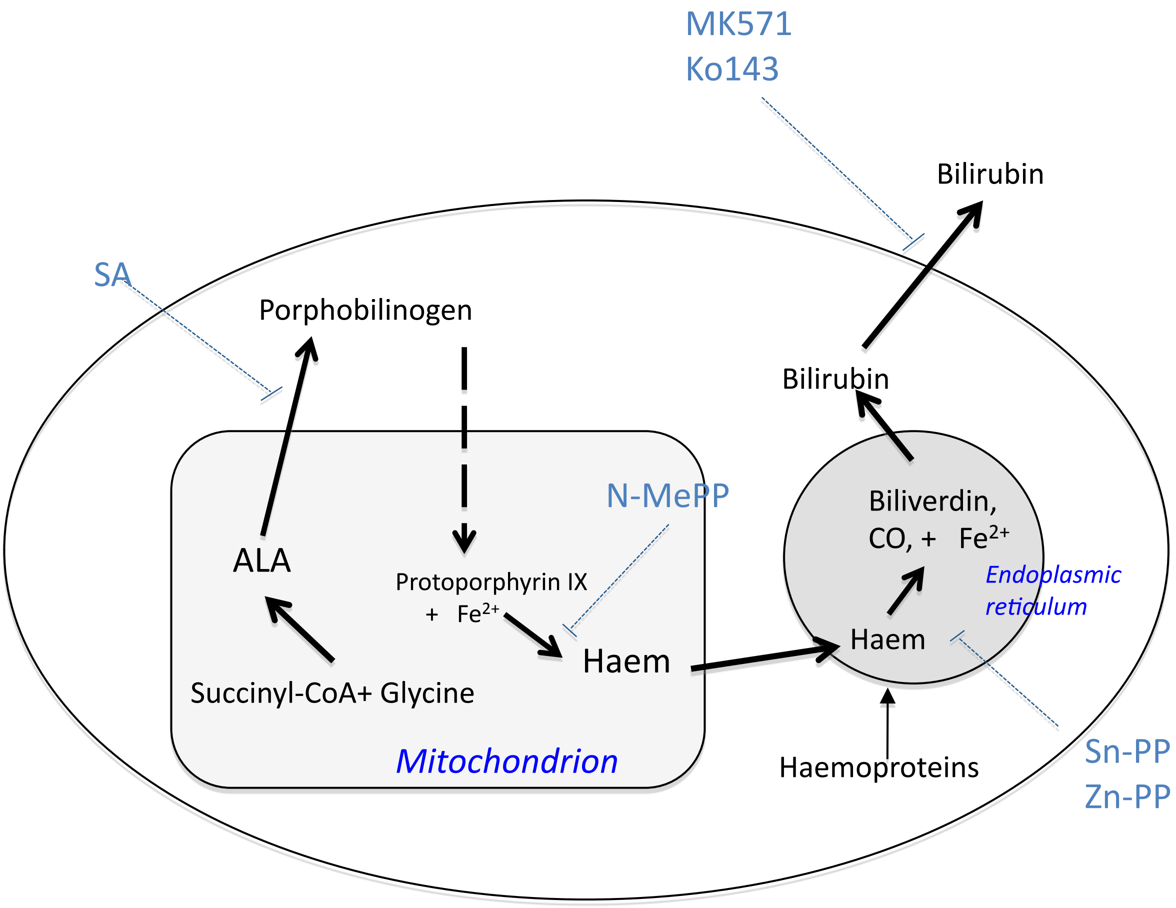

Fig. S1. Metabolism of haem and bilirubin. At the first step of haem biosynthesis, ALA is synthesised by the condensation reaction of succinyl-CoA and glycine in mitochondria. After several steps involving cytosolic enzymes, haem is finally synthesised by the insertion of ferrous ions into protoporphyrin IX in mitochondria. Haem is utilised as haemoproteins and degraded to biliverdin, CO, and ferrous ions in endoplasmic reticulum. Biliverdin is then converted to bilirubin, which is delivered outside of cells. Dashed lines indicate the inhibition sites of indicated inhibitors: SA, succinyl acetone; N-MePP, *N*-methyl protoporphyrin; Zn-PP, Zn-protoporphyrin; and Sn-PP, Sn-protoporphyrin.

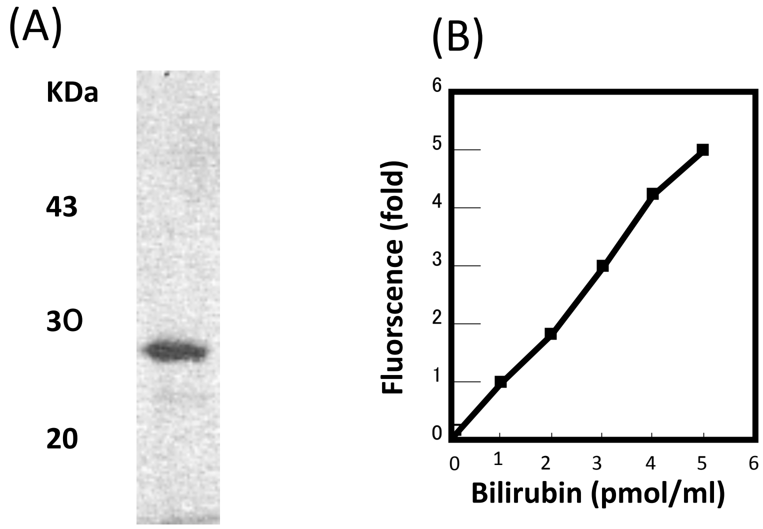

Fig. S2. Characterisation of the recombinant UnaG. UnaG was produced in *E. coli* BL21 transformed with pET-UnaG and purified using nickel ion beads after lysis of the cells. (A) Purified UnaG was separated by SDS-PAGE, and the gel was stained with Coomassie brilliant blue. (B) Binding to bilirubin. UnaG (2 μg of protein) was incubated with the indicated amount of bilirubin in VP-SFM medium at room temperature for 1 h, followed by shaking with nickel beads for 30 min. After washing the beads with TBS, UnaG was eluted with TBS containing 300 mM imidazole. The fluorescence in elutes was examined.

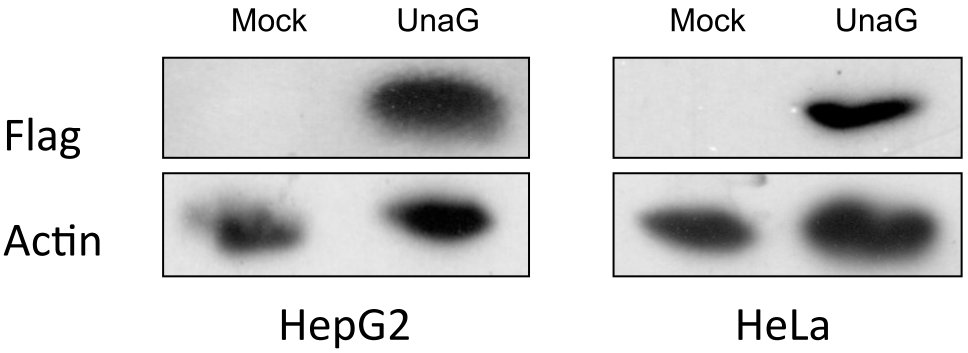

Fig. S3. Immunoblot analysis of UnaG in HepG2 and HeLa cells. The lysates of HepG2 cells and HeLa cells expressing UnaG were analysed by SDS-PAGE. After proteins were electroblotted on a PVDF membrane, the immunoblotting was performed with anti-flag and anti-actin as the primary antibodies.

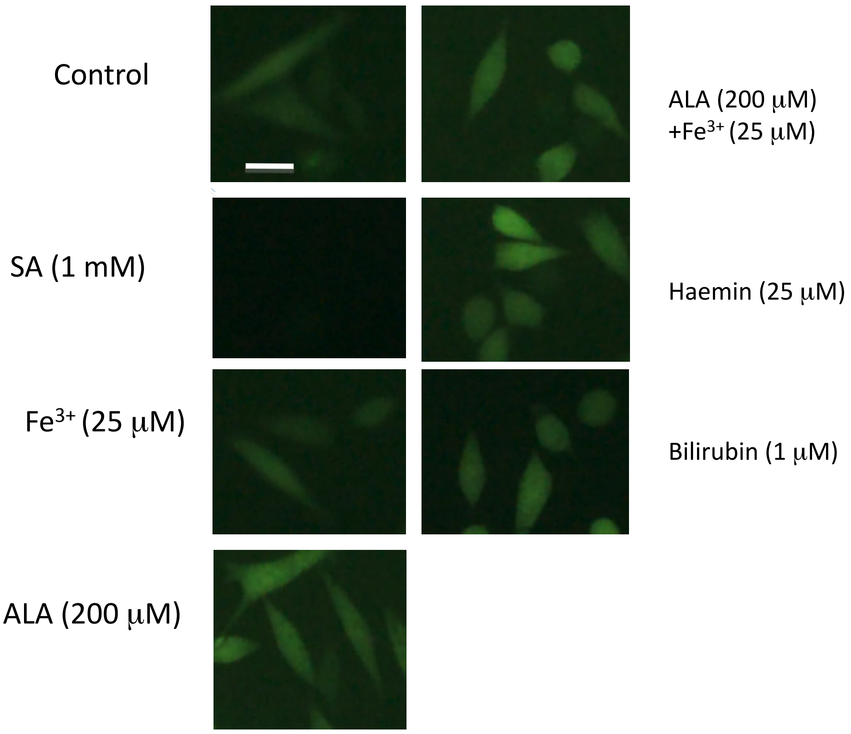

Fig. S4. Bilirubin of HeLa cells expressing UnaG. UnaG-expressing HeLa transfectants were treated with the indicated chemicals for 16 h. The level of bilirubin in the living cells was examined by fluoromicroscopy. Bar: 10 μm.
